# Impact of eptinezumab on work productivity beyond reductions in monthly migraine days: post hoc analysis of the DELIVER trial

**DOI:** 10.1186/s41687-024-00813-w

**Published:** 2024-12-18

**Authors:** Piero Barbanti, Susanne F. Awad, Heather Rae-Espinoza, Stephane A. Regnier, Xin Ying Lee, Peter J. Goadsby

**Affiliations:** 1 Headache and Pain Unit, IRCCS San Raffaele, Rome, Italy; 2 San Raffaele University, Rome, Italy; 3 H. Lundbeck A/S, Copenhagen, Denmark; 4 Clinigma, Copenhagen, Denmark; 5 California State University, Long Beach, California USA; 6 NIHR King’s Clinical Research Facility and Headache Group, King’s College, London, UK; 7 Department of Neurology, University of California, Los Angeles, California USA

**Keywords:** Absenteeism, Eptinezumab, Migraine, Presenteeism, Work productivity

## Abstract

**Background:**

Eptinezumab’s impact on self-reported work productivity in adults with migraine and 2‒4 prior preventive migraine treatment failures is not fully understood.

**Methodology:**

Electronic diaries captured monthly migraine days (MMDs) reported by patients enrolled in the randomized, double-blind, placebo-controlled DELIVER trial (NCT04418765). The migraine-specific Work Productivity and Activity Impairment questionnaire, administered at baseline and each monthly visit, was a secondary outcome of DELIVER and used to model changes from baseline in self-reported monthly hours of absenteeism (decreased work attendance) and presenteeism (reduced work efficiency while at work with a migraine) in Canada, as the base case. Path analysis illustrated eptinezumab’s impact on work productivity beyond MMDs.

**Results:**

As MMDs increased, monthly hours of absenteeism increased linearly while those of presenteeism increased quadratically. Best-fit models were improved after including an eptinezumab treatment effect, showing benefit over placebo after controlling for MMD frequency. Compared to placebo, patients treated with eptinezumab (pooled) had a modeled reduction from baseline of 7.2 h/month (absenteeism) (95% CI: −9.9, −4.5; *P* < 0.001) and 21.4 h/month (presenteeism) (95% CI: −26.3, −16.5; *P* < 0.001) over weeks 1‒24. Beyond MMD reductions, improvements in patient-identified most bothersome symptom (PI-MBS) and reductions in percent of severe migraine attacks contributed to eptinezumab’s effect.

**Conclusions:**

Eptinezumab decreased absenteeism and presenteeism based on patient reports, with data highlighting the importance of considering the PI-MBS. The greater change from baseline than placebo in self-reported absenteeism and presenteeism was only partly explained by changes in MMDs, presenting a potential opportunity to decrease the cost of migraine in the workplace.

**Trial registration:**

ClinicalTrials.gov (Identifier: NCT04418765); EudraCT (Identifier: 2019-004497-25)

**Supplementary information:**

The online version contains supplementary material available at 10.1186/s41687-024-00813-w.

## Background

Migraine is a prevalent neurological disease and recognized as a disabling medical disorder on the global level [1]. Effects are especially prominent at work, as migraine leads to substantial loss of work time due to decreased work attendance (absenteeism) and reduced work efficiency while at work with a migraine (presenteeism) [2, 3]. The societal and financial cost from lost work hours and reduced productivity are high regardless of demographics or employment status [4]. In the United States, annual total costs due to chronic and episodic migraine were $2649/year/person, respectively (2013 USD) [5]. In Canada, migraine accounted for the third-largest incremental productivity loss due to absenteeism, ~$980 million/year (2010 CAD) [6, 7]. In the UK, France, Germany, Italy, and Spain, chronic migraine was found to be associated with additional healthcare costs [7]. Across Europe, 93% of the economic burden of migraine was attributed to indirect costs such as workplace absenteeism [8].

New migraine preventive treatments, such as eptinezumab, can reduce migraine frequency and severity, thus improving work productivity and related financial burden [9, 10], even in patients who previously experienced preventive migraine treatment failures [11]. The DELIVER trial showed that patients with prior preventive failures treated with eptinezumab had larger improvements in absenteeism and presenteeism per the migraine-specific Work Productivity and Activity Impairment (WPAI:M) scale compared with patients receiving placebo [11]. There was moderate correlation between monthly migraine days (MMDs) and work productivity, as well as correlation between productivity, migraine severity, and patient-identified most bothersome symptom (PI-MBS) [11]. Similarly, health-related quality of life improvements after treatment with eptinezumab in DELIVER are driven substantially by changes in PI-MBS [12], while improvements in health utilities are also not alone explained by changes in monthly migraine days [13]. The specific pathway through which eptinezumab improves work productivity beyond MMD reduction is not well understood.

The overall aim of this subgroup analysis of DELIVER was to understand the impact of eptinezumab on work productivity levels in patients with migraine and multiple prior preventive treatment failures who were employed at baseline. To achieve that aim, several analyses were performed. The impacts of migraine on work productivity before study treatment were contextualized through qualitative analysis of exit interviews. Monthly lost work productivity and MMDs were linked and used to investigate the presence of a treatment effect on productivity, controlling for MMD frequency. Differences in monthly lost work productivity hours were compared between placebo and eptinezumab and transformed into monetary productivity gains. Finally, the potential eptinezumab treatment effect on work productivity improvement beyond MMD reduction was explained through path analysis. For primary analyses, Canada was chosen as an example because real-world treatment eligibility requirements [14] matched that of the DELIVER population, but the United States, the United Kingdom, and Germany were also analyzed to provide a more global perspective. The data were presented in preliminary form at the 75th Annual Meeting of the American Academy of Neurology (Boston, USA; April 22–27, 2023) [15].

## Methods

### Data source, study population, and definitions

DELIVER (NCT04418765) was a phase 3b, randomized, double-blind, placebo-controlled trial exploring safety and efficacy of eptinezumab in patients (N = 890) with migraine and 2‒4 prior preventive treatment failures [16]. Current subgroup analyses used data from the 28-day baseline and 24-week treatment periods of the double-blind, placebo-controlled period. Treatment was intravenously administered at day 0 and week 12.

Clinical outcomes included number of MMDs and percentage of migraine attacks with severe intensity. MMDs and associated severity were captured via daily electronic diary entries. Electronic diary reports included a daily diary (patients completed prompts each evening regardless of headache) and a headache diary (patients completed prompts for each headache). Determination of a migraine day was through yes/no responses to symptom-based questions, and severity was rated as mild, moderate or severe.

Patient-reported outcomes included the WPAI:M [17] and PI-MBS [18, 19] questionnaires. The WPAI:M was captured at baseline and at each monthly follow-up visit (assumed 4-week duration; up to six post-baseline visits), and the PI-MBS was captured at weeks 12 and 24. Both the WPAI:M and PI-MBS were captured before study drug infusion at the baseline site visit. For post-baseline visits scheduled in alignment with a site visit, the WPAI:M and PI-MBS could be completed at the site (before study drug infusion if part of site visit) or in the remote setting <3 days prior to the scheduled site visit date; for post-baseline visits scheduled to be conducted by telephone, contact had to be completed in the remote setting within 3 days prior or on the day of the scheduled telephone contact. WPAI:M absenteeism and presenteeism subscores were calculated using responses from 656 patients (Table S1) employed at baseline (eptinezumab 100 mg, n = 206; eptinezumab 300 mg, n = 223; placebo, n = 227). The absenteeism subscore was calculated by dividing Question 2 (“During the past seven days, how many hours did you miss from work because of problems associated with your migraine?”) by the sum of Question 2 and Question 4 (“During the past seven days, how many hours did you actually work?”). The presenteeism subscore was calculated by dividing Question 5 (“During the past seven days, how much did your migraine affect your productivity while you were working?”; 10-point scale) by 10. Decreases in WPAI:M subscores indicate improved work productivity. PI-MBS is a measure in which patients rate change in the most bothersome migraine-associated symptom that they had identified at baseline on a 7-point scale from 1 (“very much improved”) to 7 (“very much worse”), with 4 meaning “no change” and a decrease indicating improvement in the most bothersome symptom.

A multinational study, DELIVER recruited patients from countries with differences in average workweek length (Table S1). To generalize results to any country, absenteeism and presenteeism data were normalized to meet country-specific metrics, with Canada as the base case. Canada was chosen as the base case because treatment eligibility requirements [14] matched that of the DELIVER population. Weekly hours lost due to absenteeism and presenteeism were calculated by multiplying the respective WPAI:M subscores by the 2021 average weekly working hours of Canada (37.3 h [20]), the United States (38.7 h [21]), the United Kingdom (35.5 h [22]), and Germany (30.4 h [23]). Weekly hours were multiplied by 4 to estimate average monthly working hours.

In a sensitivity analysis using estimates of workweek hours, monthly hours lost to absenteeism were calculated by multiplying results of WPAI:M Question 2 by 4. Monthly hours lost to presenteeism were calculated by the following formula:

$$Hours_{presenteeism} = Q4 \times \frac{{Q5}}{{10}} \times 4$$

### Exit interviews

Exit interviews were conducted to better understand the perspective of patients who have unsuccessfully been treated with prior preventive treatments, their experiences with migraine prior to entering the trial, and how they balance their lives in the interictal period (i.e., the non-headache parts of a migraine cycle). Patients were eligible to participate in the exit interviews voluntarily if they had completed the randomized portion of the study and had their Week 24 assessment visit. Written informed consent to participate in the exit interview was collected at the clinical sites. The site personnel scheduled the exit interviews at a time convenient for the patients in a dedicated, secure interview platform. The interviews were conducted within 14 days after the Week 24 visit. Prior to the telephonic interviews, verbal consent from patients was also obtained. The exit interviews were conducted between January and October 2021. The semi-structured interviews were recorded and lasted up to 60 min. External, native-speaking interviewers skilled in qualitative research conducted the interviews in the patients’ own languages. Both patients and the interviewers were blinded during the interview. The patients were not offered additional compensation for participating in the interviews.

A semi-structured exit interview manual contained open ended questions pertaining to how migraine has impacted patients’ ability to participate in activities of daily life as well as coping strategies used by patients to deal with their migraines. The manual was then used to structure the discussion in the exit interviews. For example, patients were asked “Prior to the current medication, did migraines affect involvement in work or school activities in your daily life? Please explain.” Interviewers probed for “information about work or school activites such as migraines resulting in routine absenteeism, reduced productivity, or the need to manage their workload and to discuss the limitations migraine imposes.”

Exit interviews were recorded and the audio files were transcribed, coded, and analyzed by an outcomes research expert. Qualitative data analysis was conducted using Dedoose (version 9.0.46), a qualitative data management software. A continuous and comparative approach to coding was used, with an iterative engagement with transcripts to ensure consistent and reliable coding throughout. Any discrepancies were discussed and resolved. Content analysis was used to determine the impact of migraines on patients’ daily life prior to the clinical study, with key emerging themes supported by direct patient quotes. Further details on the methodology are provided in the Supporting Information—Methods.

### Relationship between work productivity and MMDs

To investigate the relationship between work productivity and MMDs independent of treatment, patient-level data were fitted to a linear model where work productivity (P; absenteeism or presenteeism) is linearly dependent on MMDs:

$${\rm{P}} = {\rm{a}} + {\rm{b}} \times {\rm{MMD}} + \epsilon$$

and to a model with an added quadratic form for MMDs:

$${\rm{P}} = {\rm{a}} + {\rm{b}} \times {\rm{MMD}} + {\rm{c}} \times MM{D^2} + \epsilon$$

Model specifications were compared to assess “best fit” using maximum likelihood models (and the *anova* function in R, a generic function used to compare the nested models), and fitted models with the lowest Bayesian Information Criteria were chosen as best-fit models. Given the repeated measures over the 24-week period in DELIVER (one measure/month), these models included up to six observations per individual (including baseline MMD values).

To determine the relationship between work productivity, number of MMDs, and any additional treatment effect independent of MMD reduction, the trial treatment received was added as a covariate to the best-fit models from above. The updated model excluded the baseline value since the treatment effect would not yet have materialized at baseline (therefore included up to five observations/patient).

Work productivity differences at baseline between eptinezumab and placebo arms were tested using a *t*-test to ensure that any potential treatment-specific results were not driven by imbalances between arms. To account for repeated measures per individual in the models with and without treatment effect, mixed linear models were used with random effect for individuals.

### Treatment effect on work productivity through MMD frequency, migraine severity, and PI-MBS

To estimate the change from baseline and differences in monthly productivity hours between placebo and eptinezumab, the mean change from baseline was estimated for placebo, eptinezumab 100 mg, eptinezumab 300 mg, pooled eptinezumab arms, and treatment responders (i.e., patients with ≥50% reduction in MMDs over weeks 1‒12) in the pooled eptinezumab arms. The responder subgroup was included to evaluate the impact of confirmed response on work productivity. Mean differences from placebo and 95% confidence intervals were calculated in the full analysis set and patient subgroup with high-frequency episodic migraine or chronic migraine. The full analysis set included all randomized and treated patients with ≥1 valid 4-week assessment of MMDs during weeks 1‒12. High-frequency episodic migraine (≤14 monthly headache days including 8‒14 MMDs) and chronic migraine (≥15 monthly headache days including ≥8 MMDs) were based on the daily electronic diary reports during the 28-day baseline period.

MMDs were calculated as the number of migraine days within each 28-day interval using the imputation rules as previously published [16]. Analyses were made based on complete cases (only patients with valid variable scores at corresponding time points) and no attempts were made to account for missing data beyond MMD calculations. Change from baseline estimates were derived from a mixed model for repeated measures (MMRM), which included the following fixed effects: visit (study month), country, stratification factor (monthly headache days at baseline: ≤14/>14), and treatment as factors; baseline score (hours) as a continuous covariate; and baseline score-by-visit interaction, treatment-by-visit interaction, and stratum-by-visit interaction (stratum being a predefined partition of the overall study population). The MMRM is estimated via a linear model using generalized least-squares with an unstructured covariance matrix for the vector of repeated measures for each patient (change from baseline differences in absenteeism and presenteeism for eptinezumab vs placebo).

To explain the potential treatment effect on work productivity improvement beyond MMD reduction, path analyses were generated which estimated *direct effect* (i.e., the effect of eptinezumab vs placebo on work productivity not explained through its effect on migraine-related measures) and *indirect effect via a mediator*. In a model where change in MMDs is the only mediator, the indirect effect represents the eptinezumab impact on work productivity explained by its impact on MMDs, which in turn improves work productivity (Fig. S1). An entirely indirect effect indicates a good biological understanding of the treatment effect. Four paths (i.e., Models 1‒4) were created to better explain the effect of eptinezumab on work productivity. All paths included change in MMDs as a mediator, and Models 2–4 varied in including changes in percentage of post-baseline migraine attacks with severe pain intensity (Model 2), changes in PI-MBS (Model 3), or both (Model 4) as additional mediators.

All time points across weeks 1‒24 were used in the path analysis (accounting for potential correlations for observations for the same patients using the cluster command). For Models 3 and 4, there was a maximum of two data points/patient (weeks 12 and 24) because change in PI-MBS was only collected at two time points. Consequently, only WPAI:M and MMD data at the same time points were used in this path analysis.

### Cost savings of improved work productivity

To quantify monthly monetary benefits, the hours/month of productivity gained were multiplied by the 2021 average hourly wage rates in Canada (base-case; 33.22 CAD [24]), the United States (28.01 USD [25]), the United Kingdom (14.48 GBP [26]), and Germany (23.56 EUR [27]).

### Software and coding

For each fitted model for the relationship between monthly work productivity and MMDs, coefficients were estimated via restricted maximum likelihood with the *lmer* function from the *lme4* R package v4.2.2. To find the best-fit model, the *anova* function in R was used. The MMRM was estimated using the *gls* function from the R package *nmle* with an unstructured correlation matrix and distinct variance for each follow-up visit. Path analyses were conducted using the *lavaan* package in R. Significance was assessed at *P* < 0.05. For the exit interviews, coding of data was done using Dedoose (v9.0.46).

## Results

### Exit interviews

Based on exit interviews, patients described migraine’s impact on work productivity/performance, career opportunities/growth, and employment (Fig. S2). Of the first 345 randomized patients who completed the randomized portion of the study, 100 patients consented to participate in exit interviews. At the time of the interviews, 3 of the 100 patients who had consented to participate were lost to follow-up, and the final exit interview subgroup comprised 97 patients (at sites in the United States, France, Denmark, Italy, Spain, Germany, and the United Kingdom). WPAI:M scores were obtained from 73 of the exit interview subset participants.

Most patients interviewed (92/97, 95%) reported on how migraine affected their work activities. Among these 92 patients, the majority (79/92, 86%) described a *negative impact on work productivity/performance*, such as one participant indicating “not being able to perform at the level [I wanted to]…or not being able to respond to certain demands.” Reduced concentration impacted quality and timely completion of tasks, leading to feelings of inadequacy. Over half (57%) *endured pain to finish work*, or “suffered in silence,” as one patient described. Despite “constant limiting pain,” many patients perceived no choice to pause/skip work, contributing to presenteeism. Migraine attack frequency and/or intensity led to changes from full- to part-time employment, and in extreme cases, quitting. As one respondent stated, “I’ve had other jobs where I put in a 2-week notice just because it was starting to get to the point where I was…missing so many days of work that I knew that my job was suffering from it. I just don’t like being a burden on people.” Most (68%) experienced *limited functionality* and consequently lost income due to reduced hours and excessive sick leave. Reduced work productivity due to migraine was common in this population, with interviewees perceiving limited control over work attendance and workload, leading to negative emotional and financial outcomes.

### Relationship between work productivity and MMDs

**Fig. 1:**
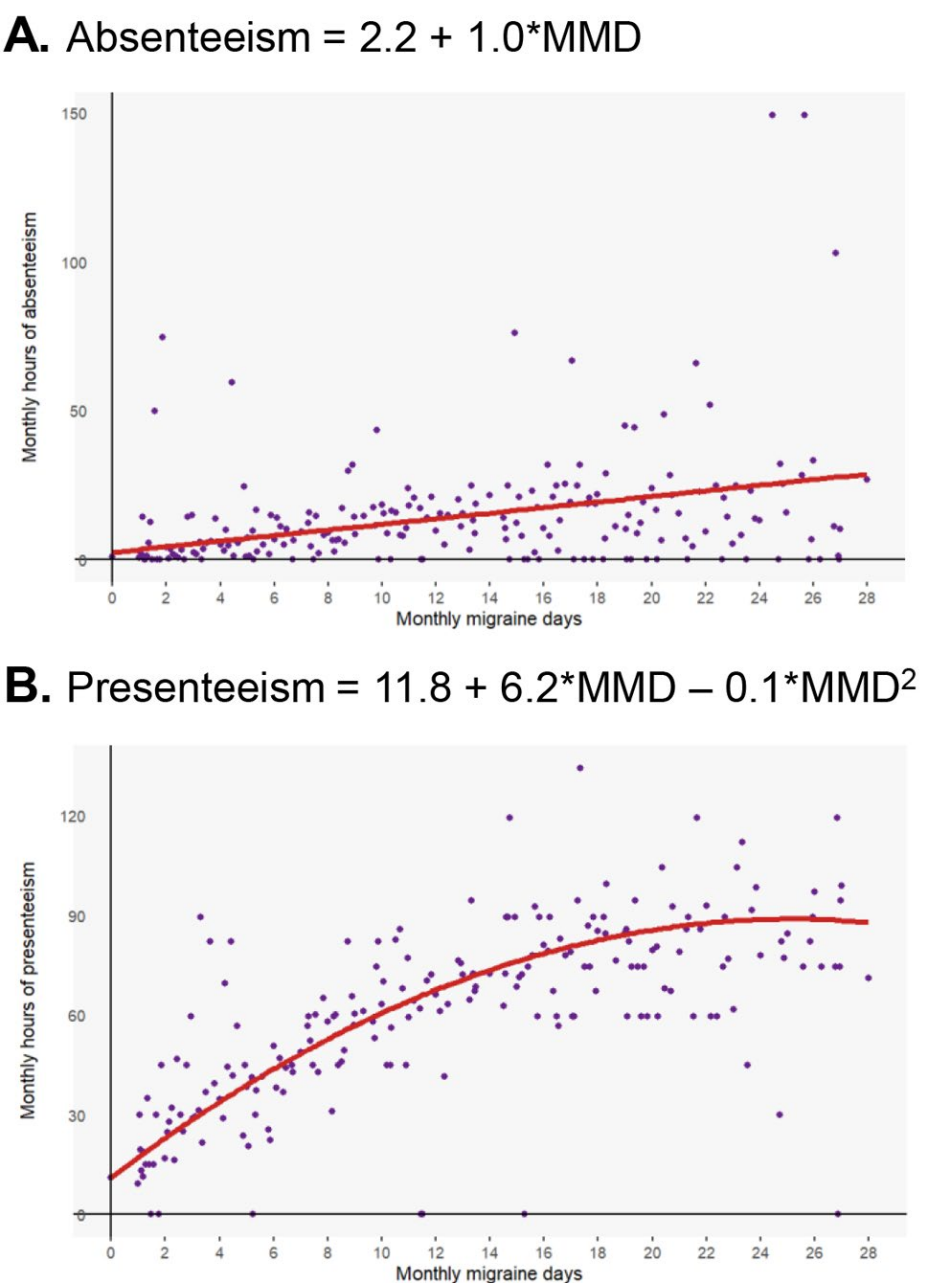
Relationship between MMDs and monthly hours of **A** absenteeism and **B** presenteeism. Includes eptinezumab and placebo arms pooled. Number of observations from patients (*N* = 656): absenteeism, 3847; presenteeism, 3793. Note that several points may appear hidden because they represent the same pairwise (x, y) values as other points. *MMDs* monthly migraine days

Based on best-fit assessment (Tables S2 and S3), there was a linear relationship between MMDs and absenteeism (Fig. 1A) and a quadratic relationship between MMDs and presenteeism (Fig. 1B). In the absenteeism linear model, higher MMDs were associated with more work hours missed. An additional MMD represented an average increase in monthly absenteeism of 1.0 hour (95% CI: 0.9, 1.2). The average difference in monthly absenteeism was ~29 h between 0 and 28 MMDs (i.e., 28 MMDs was associated with ~29 more hours of absenteeism than 0 MMDs), representing ~3.6 workdays/month assuming an 8-h workday. Many patients (286/656 [44%]) reported not missing any workdays. In the presenteeism quadratic model, the more MMDs, the more work hours with reduced productivity, up to 25 MMDs; after 25 MMDs, the curve straightened as saturation was reached. The average difference in monthly presenteeism was ~78 h between 0 and 28 MMDs (i.e., 28 MMDs was associated with ~78 more hours of presenteeism than 0 MMDs), representing ~9.8 workdays/month (assuming an 8-hour workday).

### Eptinezumab treatment effect on work productivity

For almost all frequency groups of MMD values (i.e., MMDs were grouped into equally sized buckets including 0– <2, 2– <4, etc.), absenteeism and presenteeism hours were higher for patients receiving placebo versus eptinezumab (Fig. S3), motivating inclusion of treatment effect in the models (Table S4). Eptinezumab-treated patients had on average 4.8 fewer hours/month of absenteeism and 10.8 fewer hours/month of presenteeism than did patients receiving placebo with similar numbers of MMDs.

On average during weeks 1‒24, eptinezumab-treated patients experienced a substantial improvement in presenteeism compared to patients receiving placebo (Table 1). In Canada, compared to baseline values, patients on eptinezumab (pooled) had a reduction in absenteeism and presenteeism of 8.3 and 33.4 h/month, respectively, which was 7.2 h/month less work absence and 21.4 h/month more efficiency while at work versus placebo. Similar results across arms were observed for patients with high-frequency episodic migraine or chronic migraine (Table S5). Differences from placebo in change from baseline in absenteeism and presenteeism with eptinezumab (pooled) were modeled to be −7.5 and −22.2 h/month, respectively, in the United States (Table S6), −6.8 and −20.2 h/month in the United Kingdom (Table S7), and −5.9 and −17.4 h/month in Germany (Table S8; *P* < 0.001 for all comparisons). In the sensitivity analysis using DELIVER-derived estimates, differences from placebo with eptinezumab (pooled) were −5.2 and −17.3 h/month, respectively, consistent with the base case (Table S9).

**Table 1:** Analysis of monthly absenteeism hours and presenteeism hours, using Canadian workweek data, for weeks 1‒24

Treatment	Baseline		Change from baseline		Comparison to placebo		
	***N***	Mean (SD)	N^†^	Mean (SE)	Difference	95% CI	*p*-value
**Absenteeism**^**‡**^							
Placebo	218	19.2 (29.9)	210	−1.0 (1.73)			
Eptinezumab 100 mg	196	17.0 (28.9)	192	−8.4 (1.76)	−7.4	(−10.6, −4.3)	<0.001
Eptinezumab 300 mg	209	17.8 (28.8)	204	−8.0 (1.69)	−7.0	(−10.1, −3.9)	<0.001
Eptinezumab pooled	405	17.4 (28.8)	396	−8.3 (1.52)	−7.2	(−9.9, −4.5)	<0.001
Eptinezumab responders^§^	185	17.5 (27.3)	182	−13.0 (1.75)	−11.7	(−14.8, −8.5)	<0.001
**Presenteeism**^**¶**^							
Placebo	212	77.1 (36.1)	206	−12.2 (3.16)			
Eptinezumab 100 mg	191	75.8 (38.2)	187	−33.8 (3.20)	−21.6	(−27.4, −15.8)	<0.001
Eptinezumab 300 mg	206	79.6 (35.8)	203	−33.4 (3.08)	−21.2	(−26.9, −15.6)	<0.001
Eptinezumab pooled	397	77.8 (37.0)	390	−33.4 (2.77)	−21.4	(−26.3, −16.5)	<0.001
Eptinezumab responders^§^	183	77.6 (39.9)	181	−50.2 (2.99)	−37.2	(−42.6, −31.8)	<0.001

*CI* confidence interval, *MMDs* monthly migraine days, *SD* standard deviation, *SE* standard error. Analysis was of the full analysis set and utilized a mixed model for repeated measures

^**†**^Number of patients with ≥1 measurement

^**‡**^3088 observations

^§^Responders are defined as patients reaching ≥50% reduction from baseline in MMDs for each month in the 3-month interval (weeks 1‒12)

^**¶**^3005 observations

### Potential cost savings of improved work productivity with eptinezumab

**Fig. 2:**
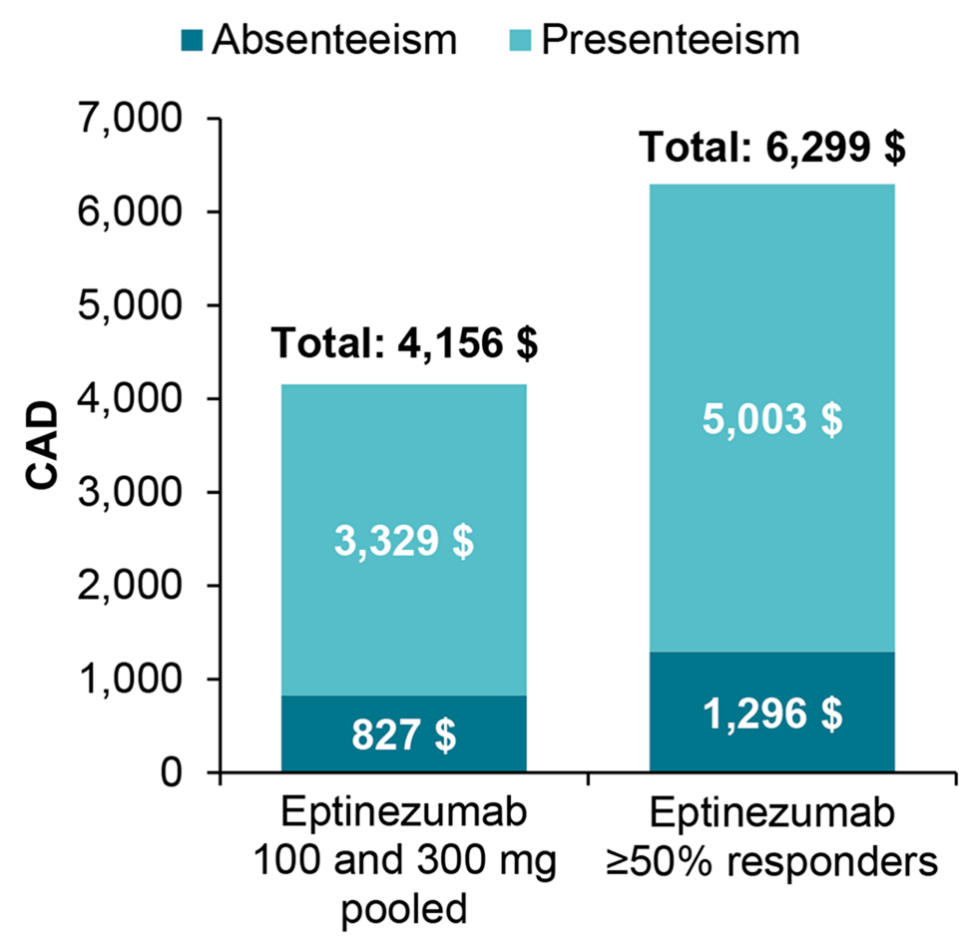
Value of productivity gains following one eptinezumab infusion over 12 weeks (3 months) in Canada. Value of productivity gains = change from baseline in monthly hours in pooled eptinezumab group × 3 months × 33.22 CAD. The costing hours for patients (N = 656) affected by presenteeism were assumed to be at a 100% productivity level. *CAD* Canadian dollars

Eptinezumab (pooled) gained patients an estimated ~4156 CAD in productivity over 3 months (Fig. 2). Savings for other countries are in Fig. S4.

### Eptinezumab effect on work productivity improvement beyond MMD reductions

**Fig. 3:**
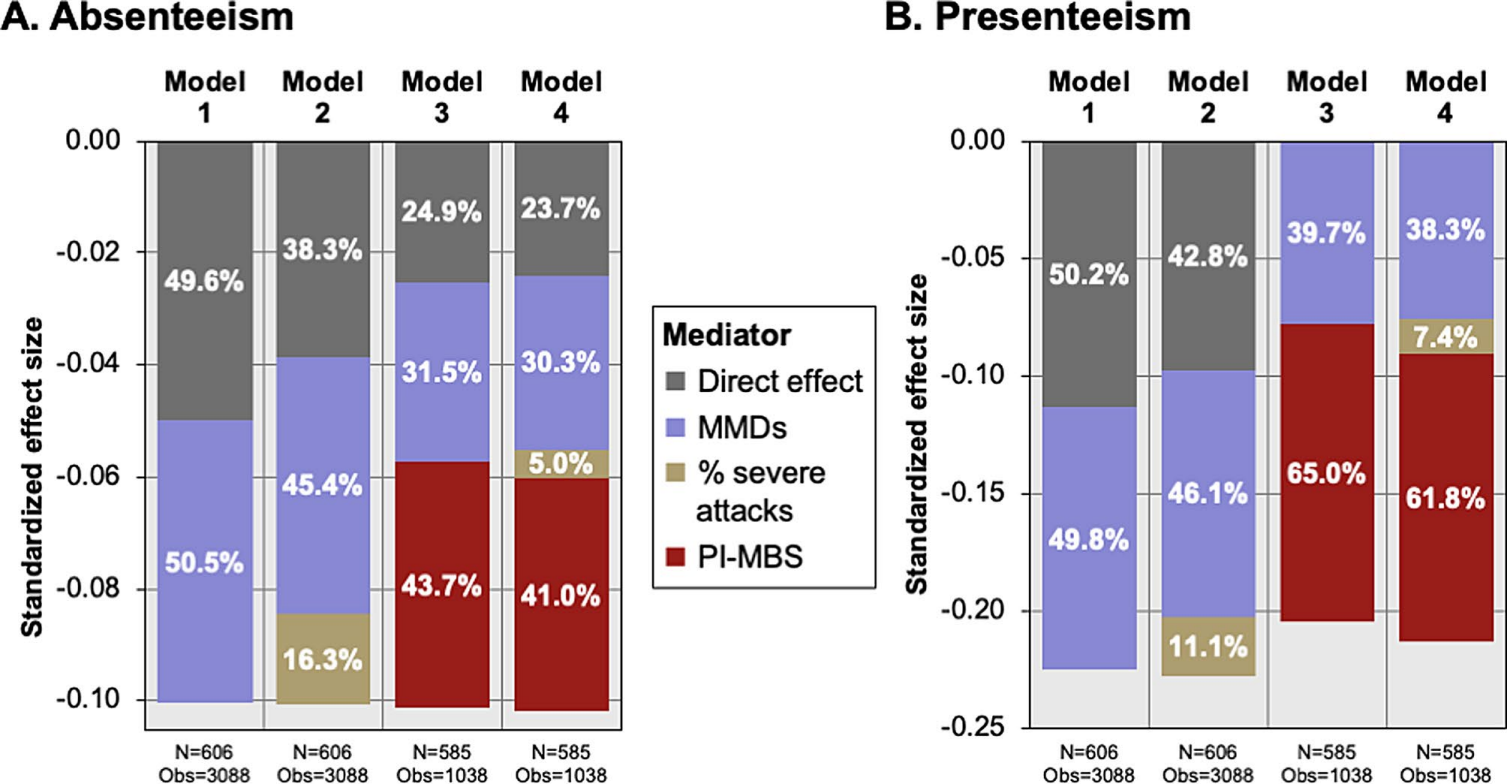
Direct versus indirect treatment effect for **A** absenteeism and **B** presenteeism. Due to rounding, some percentages may not add up to 100%. For presenteeism Models 3 and 4: Please see Fig. S7 for these results with the direct effect included. The direct effect was small (<5%), negative, and not significant (*P*-value <0.05) meaning that the direct effect could not be proven to be different from 0 and the sign of the coefficient should not be overly interpreted. The indirect effects fully account for the relationship between the treatment and the outcome, resulting inan estimated direct effect that could be influenced by random fluctuations and lead to an artificially small or even negative value. MMDs, monthly migraine days; PI-MBS, patient-identified most bothersome symptom.

With change in MMDs as a sole mediator of treatment effect (Model 1), path analysis of the impact on absenteeism found that eptinezumab reduced MMDs by 3.14 days relative to placebo, with each MMD associated with an increase of 1.09 absenteeism hours/month compared to placebo (Fig. S5A). Thus, eptinezumab reduced absenteeism indirectly by 3.42 h/month via MMD reduction (i.e., −3.14 × 1.09). The direct effect reduced absenteeism by 3.35 h/month, hence comprising 49.6% of the total effect on absenteeism (Fig. 3A). Model 2 reduced the direct effect to ~38%, with ~16% of the effect of eptinezumab on improvement in absenteeism hours/month associated with change in severe migraine attacks (Figs. 3A and S5B); Model 3 reduced the direct effect to ~25%, with ~44% of the effect of eptinezumab on improvement in absenteeism hours/month associated with change in PI-MBS (Figs. 3A and S5C). Model 4 results were like Model 3, with change in severe migraine attacks accounting for only 5% of the eptinezumab effect on improvement in absenteeism hours/month (Figs. 3A and S5D).

In the Model 1 path diagram analyzing impact on presenteeism, the direct effect of eptinezumab was −11.26 h/month (Fig. S6A), comprising 50% of the total effect in presenteeism improvement (Fig. 3B). The direct effect was slightly reduced (43%) after adding migraine severity as a mediator (Model 2; Figs. 3B and S6B). Including PI-MBS (Model 3), the direct effect diminished completely (Fig. 3B), and the effect of improvement in PI-MBS was attributed to 64% of improvement in monthly presenteeism hours/month (Figs. S6C and S7). Model 4 results (all variables) were similar to those of Model 3 (Fig. 3B), with change in severe migraine attacks accounting for only 7% of the eptinezumab effect on improvement in presenteeism hours/month (Figs. S6D and S7).

## Discussion

Patients in DELIVER indicated that migraine had a substantial negative impact on work life, including work productivity, career opportunities, and employment. Exit interviews revealed that inability to consistently communicate, concentrate, perform, or even show up to work during a migraine attack led to negative emotional and financial outcomes and reduced quality of life. Annually, patients with migraine miss work on average 4.4 days and go to work with reduced productivity on average 11.4 days [28]. Extrapolating DELIVER baseline to annual rates, the eptinezumab (pooled) and placebo groups missed ~26 and ~29 workdays on average, respectively, and had ~117 and ~116 days of lost productivity, respectively. Among patients with prior treatment failures, work impairment was usually greater compared to treatment-naïve patients [29]. The eptinezumab effect as modeled here is particularly valuable information for difficult-to-treat populations such as that of the DELIVER trial.

Patients’ improvement in work productivity was comparable between eptinezumab doses and represented a presenteeism improvement of 33 h/month for eptinezumab (pooled), or approximately 4 workdays/month, or ~43% (33.4/77.8) relative to baseline. Improvements were higher for eptinezumab-treated patients with ≥50% MMD reduction over weeks 1‒12 (reduction of 50.2 h/month on average, or ~6 workdays/month, or 65% [50.2/77.6]). In previous linear regression models examining work productivity of European patients with migraine, every 1-day increase in headache-free days (among the general migraine population) led to an average presenteeism decrease of 2.1% [30]. In comparison, this work proposes a quadratic relationship between MMDs and presenteeism hours/month in patients with 2–4 prior migraine preventive treatment failures. Results here suggest that every 1-day reduction in MMDs led to a 7.9% average decrease in presenteeism hours. The quadratic relationship suggests a cumulative effect of migraine on presenteeism in this population. The rapid and sustained reduction in MMDs reported in previous trials [9, 16, 31–34] also suggest that treatment with eptinezumab may be beneficial for work productivity and efficiency in patients within a difficult-to-treat migraine population.

Path analyses assessed direct and indirect effects of migraine treatment on work productivity. When patients describe complicated trial-and-error experiences with managing the various dimensions of migraine, path analysis and modeling of clinical trial data can help clarify how overlapping effects shape a difficult-to-treat disorder. Change in MMDs as a sole mediator of treatment effect explained approximately half of the eptinezumab effect for both absenteeism and presenteeism, meaning that MMD reductions were not the only biological reason for work productivity improvement. An entirely indirect effect would suggest a biological understanding of the treatment effect, implying that the pathways are well understood and captured by the mediator (MMDs). However, the partial mediation observed here could include direct effects of eptinezumab on other symptoms of migraine, such as intensity or duration, which may not be fully accounted for by changes in MMD alone. The impact of eptinezumab on percentage of migraine attacks with severe pain intensity contributed to a lesser degree, but the combination of change in MMDs and change in PI-MBS (Model 3) explained the much greater improvement with eptinezumab versus placebo for absenteeism (~75% of the effect) and presenteeism (~100% of the effect). This is consistent with PI-MBS being closely correlated to treatment efficacy and migraine burden [18]. Previous work showed that indirect costs can substantially outpace direct costs [35] and that migraine severity placed a noticeable burden on healthcare systems [35, 36]. Future work on the interictal period may further explain indirect effects of migraine.

Path analyses that examine mediators of treatment efficacy help payors understand how eptinezumab impacts the career progression of patients with migraine. Patients might be forced to work part-time to accommodate symptoms [37], as echoed by DELIVER interviewees (“I went to my part-time job, mainly because of migraines”), or miss work to schedule frequent visits with healthcare providers [36]. Interviewees experienced limited functionality, resulting in income losses due to reduced hours, excessive sick leave, and ultimately job loss. Future work should explore the threshold at which treatments targeting calcitonin gene-related peptide start offsetting treatment costs in terms of productivity gains. Monetarized improvements with eptinezumab in absenteeism and presenteeism hours/month equate to over 4000 CAD. Beyond economic value, there is a greater personal loss for patients associated with work productivity, since managers might not promote workers with migraine who frequently miss work [38], as suggested by DELIVER interviewees. Work productivity levels were also related to self-esteem, not just financial compensation, and were closely tied to a sense of freedom, an effect that is not captured in the WPAI:M. An effective migraine preventive treatment that alleviates migraine pain severity and migraine-related symptoms, while reducing migraine frequency, may bring about improvement to multiple factors behind work productivity.

### Limitations

The DELIVER population was primarily White and female [16], limiting generalizability, and these analyses were limited to currently employed patients, which may have excluded individuals with more severe disease who exited the workforce. Some patients may have already adjusted their schedules to accommodate migraine, which can lower response rates [29] and could have affected the total possible work hours. The path analysis excluded patients with missing model variables (aside from MMDs) at any timepoint. For modeling with continuous variables, it was assumed that ordinal responses to the WPAI:M were linear/proportional, and the simplest model was chosen. Additional types of impact (e.g., educational and career progression) are not captured in the WPAI:M data, and thus the analysis can be seen as conservative. The interviews were captured in the employed DELIVER study population, which may limit generalizability.

Country-specific results were based on the same underlying data from DELIVER; however, the impact of eptinezumab treatment may not be the same for every country, and no patients from Canada were enrolled in DELIVER. It was investigated if the WPAI:M improvement observed in DELIVER varied by the patients’ country of residence. Thus, the Akaike Information Criteria (AIC) and Bayesian Information Criteria (BIC) were compared for MMRM models with and without the patients’ country of residence as a covariate (Table S10). Based on the AIC and BIC values assessing model fit to the data, the MMRM model with a country effect had a lower AIC, suggesting that it might be the better model fit according to this criterion. However, the MMRM model without a country effect had a lower BIC, suggesting that it might be the simpler model which fits the data. Given that the BIC tends to penalize more complex models than the AIC, these results suggested that the MMRM model including a country effect was a better fit to the data and therefore, we could not rule out the possibility of eptinezumab’s treatment effect on presenteeism and absenteeism varying by the patient’s country of residence. However, the BIC results also suggested there was a chance that the model including a country effect could be overfitting the data, and thus further work is needed.

## Conclusions

Patients with migraine and 2‒4 prior preventive treatment failures reported a varied and substantial negative impact of migraine on work productivity prior to treatment in the DELIVER study of eptinezumab. In the subgroup of patients in the DELIVER trial who were employed at baseline, modeling showed that as MMDs increased, monthly absenteeism increased linearly while monthly presenteeism increased quadratically. The best-fit models were improved with the inclusion of a treatment effect controlling for MMDs and showed that eptinezumab-treated patients had larger reductions in self-reported work productivity (absenteeism and presenteeism) relative to baseline compared to patients receiving placebo. The monetarized reductions in absenteeism and presenteeism suggest that the potential productivity gains with eptinezumab could have substantial economic implications, such as offsetting the price of the drug. The eptinezumab effect on work productivity was largely attributable to changes in PI-MBS and to percent of migraine attacks with severe pain intensity, in addition to reductions in MMDs.

## Electronic supplementary material

Below is the link to the electronic supplementary material.

Supplementary Material 1

## Data Availability

In accordance with EFPIA’s and PhRMA’s “Principles for Responsible Clinical Trial Data Sharing” guidelines, Lundbeck is committed to responsible sharing of clinical trial data in a manner that is consistent with safeguarding the privacy of patients, respecting the integrity of national regulatory systems, and protecting the intellectual property of the sponsor. The protection of intellectual property ensures continued research and innovation in the pharmaceutical industry. Deidentified data are available to those whose request has been reviewed and approved through an application submitted to https://www.lundbeck.com/global/our-science/clinical-data-sharing.

## Abbreviations

PI-MBS: Patient-identified most bothersome symptom
MMDs: Monthly migraine days
MMRM: Mixed model for repeated measures
WPAI:M: Migraine-specific Work Productivity and Activity Impairment
