# Supplementary material for: Impact of eptinezumab on work productivity beyond reductions in monthly migraine days: post hoc analysis of the DELIVER trial
Source: J Patient Rep Outcomes. 2024 Dec 18;8:146. doi: 10.1186/s41687-024-00813-w (PMC11655748; doi:10.1186/s41687-024-00813-w)
Supplement: Supplementary file 1 — Supplementary Material 1 [file 41687_2024_813_MOESM1_ESM.docx]

**SUPPORTING INFORMATION**

**Methods**

*Qualitative Systematic Review*

A systematic literature review, following PRISMA guidelines, was conducted to understand episodic and chronic migraine and how migraine impacts patients and their families, and to construct the initial cognitive interviews with patients to create the semi-structured interview guide. Searches were conducted using MEDLINE, EMBASE, and Cochrane Library bibliographic databases, with the last search in March 2020. These searches were supplemented with a citation analysis process in which systematic reviews on the topic were analyzed for studies citing or cited by the review, to ensure comprehensive retrieval.

Eligible studies explored the quality of life and burden of disease for adult patients with migraine (with or without aura, episodic or chronic, medication overuse migraine), used qualitative methods, and were published in English as full text in peer-reviewed academic journals within the last 10 years. Quantitative studies that focus on economic and financial costs facing patients with migraine were included, excluding those that explore wider costs to societies and economies. Research investigating the development of migraine patient-reported outcome measures were included if they contained data satisfying the above eligibility criteria. The burden on patient quality of life was defined broadly; studies were included if they examined any aspect of the patient’s work life, social life, education, activities, emotions, mental health, finances, physical abilities, or experience of symptoms.

After the completion of searches, titles and abstracts were screened for relevancy by two reviewers (DR, HRE). Full-text articles were retrieved and independently assessed against the above eligibility criteria by the two reviewers, with inclusion and exclusion agreed by consensus after discussion. Included studies were assessed for methodological quality by both reviewers using the CASP qualitative critical appraisal checklist.^1^ Fourteen studies were included in the synthesis.

*Patient Interviews*

Prior to the interviews, a semi-structured interview manual—informed by procedures to assure content validity following the U.S. Food and Drug Administration (FDA) Patient-Focused Drug Development Guidance series (Guidance 2),^2^ expert consultation, and cognitive interviews—was developed to guide the interviews, which were conducted telephonically, recorded, then transcribed verbatim.

*Coding of Exit Interview Data*

For content analysis, a codebook was developed to guide data extraction, and both inductive and deductive coding approaches were applied across cycles. Three separate but interlinked cycles of coding per Saldaña et al.^3^ were used as a framework to maximize the depth and breadth of detail garnered from each transcript. Cycle 1 data were deductively coded using predefined item-level concepts specific to the interview manual. The interview manual was informed by the systematic literature review and other materials as described in the data collection section. During Cycle 2, data were deductively coded using a predetermined theoretical framework informed by the qualitative systematic review. An adapted version of the Mannix et al.^4^ “Migraine Disease Model – Subjective Experiences of Migraine – EM and CM” was utilized, which covers the themes on burden of disease. Cycle 3 data were inductively coded using an emergent open-coding method adopted to highlight how patients’ history of migraine impacted context, daily lives, and health-related quality of life. Additionally, thematic analysis was applied to Cycle 3 coded data. Researchers read each excerpt and applied descriptive codes relating to the overarching study objectives. As in Cycles 1 and 2, following coding, the independent researchers collaborated to determine and define higher ordinate themes that faithfully captured both the qualitative richness of extreme cases and variability across participants’ migraine experiences.

Coding of data was done using Dedoose (Version 9.0.46), a qualitative data management software program. The software was designed for analysis of textual data. This allowed researchers to preload Cycle 1 and Cycle 2 coding deductive frameworks.

To assess reliability, raw data were exported from Dedoose onto an MS Excel spreadsheet. The Excel outputs from each researcher who independently coded each transcript were merged and 10% of the excerpts in each code were randomly selected. These codes and accompanying excerpts were used to determine Gwet Agreement Coefficient 1 (AC1).^5^ Data were analyzed using Stata/SE 17.0 with the resultant output.

Overall, the inter-rater reliability suggests that the researchers were reliable at identifying participants’ insights in relation to the identified codes in the coding dictionary. With 10% of double-coding assessed, researchers agreed 80.4%. Then, any discrepancies in the entire dataset were resolved. The inter-rater reliability for initial coding instances was good (AC1 = 0.76). Due to limited resources, time, and a large data set we were unable to re-run inter-coder reliability assessments. However, all discrepancies were reviewed and resolved through discussion by the research team and the research lead.

In addition, to maintain data quality, we adopted the approach proposed by the FDA (Guidance 2)^2^ on methods to identify what is important to patients. We consistently applied an iterative process of coding transcripts, we ensured that codes are applied consistently to all data by inter-rater coding, and we further examined agreement among multiple coders to avoid inconsistent coding.

The analysis for Cycle 1 and Cycle 2 included exporting all coded raw data onto an MS Excel spreadsheet. We further generated a comprehensive list of all excerpts under each code. We achieved this by merging all excerpts generated from coding framework for each code into individualized spreadsheets. There were three analysis teams, and each pair was assigned data to analyze. Excerpts were read and broken down to make meanings. An iterative process of re-reading, re-adjusting, and re-coding was conducted until each team was happy with the final subordinate codes. In line with content analysis, each occurrence of a subordinate code was dichotomized (1 = Yes, 0 = No). Once all data had been analyzed, two researchers reviewed the subordinate codes to generate superordinate themes that described participants’ experiences and were aligned with the interview schedule questions. The superordinate themes were reviewed by the team of researchers and the lead researcher. Once approved, the subordinate codes were tallied and subsumed into their corresponding superordinate themes. Frequency counts per theme were then compared with final sample to determine proportions.

Thematic analysis was applied to Cycle 3 coded data. Researchers read each excerpt and applied descriptive codes relating to the overarching study objectives. As in Cycles 1 and 2, following coding, the independent researchers collaborated to determine and define higher ordinate themes that faithfully captured both the qualitative richness of extreme cases and variability across participants’ migraine experiences.

Quantitative data from the scale questions were analyzed using Stata/SE 17.0. Descriptive statistics were utilized.

**References**

1. Critical Appraisal Skills Programme. CASP Qualitative Studies Checklist. CASP Checklists. 2018. Accessed: May 2, 2023. Available from: <https://casp-uk.net/casp-tools-checklists/>.
2. U.S. Food and Drug Administration. Patient-Focused Drug Development: Methods to Identify What Is Important to Patients: Guidance for Industry, Food and Drug Administration Staff, and Other Stakeholders. Released: February 2022. Accessed March 23, 2023. Available from: <https://www.fda.gov/media/131230/download>.
3. Saldaña JM. *The Coding Manual for Qualitative Researchers*. 2nd ed. Sage; 2012.
4. Mannix S, Skalicky A, Buse DC, et al. Measuring the impact of migraine for evaluating outcomes of preventive treatments for migraine headaches. *Health Qual Life Outcomes*. 2016;14(1):143.
5. Gwet KL. Computing inter-rater reliability and its variance in the presence of high agreement. *Br J Math Stat Psychol*. 2008;61(Pt 1):29-48.

**Table S1.** Weekly working hours by country, derived from WPAI:M Question 4 in DELIVER^†^

| **Country** | **Weekly working hours (average)** | ***N* patients** |
| --- | --- | --- |
| Poland | 33.8 | 204 |
| Czech Republic | 30.9 | 158 |
| Georgia | 35.8 | 112 |
| Slovakia | 27.7 | 22 |
| Bulgaria | 32.0 | 21 |
| Germany | 21.9 | 19 |
| France | 33.1 | 17 |
| Belgium | 27.9 | 16 |
| United Kingdom | 23.0 | 16 |
| Hungary | 29.7 | 16 |
| Spain | 34.9 | 15 |
| Russia | 37.2 | 11 |
| Denmark | 20.1 | 10 |
| Finland | 20.3 | 10 |
| United States | 23.6 | 5 |
| Italy | 13.0 | 2 |
| Sweden | 39.5 | 2 |

^†^Question 4 was, “During the past seven days, how many hours did you actually work?”

WPAI:M, migraine-specific Work Productivity and Activity Impairment.

**Figure S1.** Path analysis illustrative example showing impacts of effects on the outcome, work productivity

**
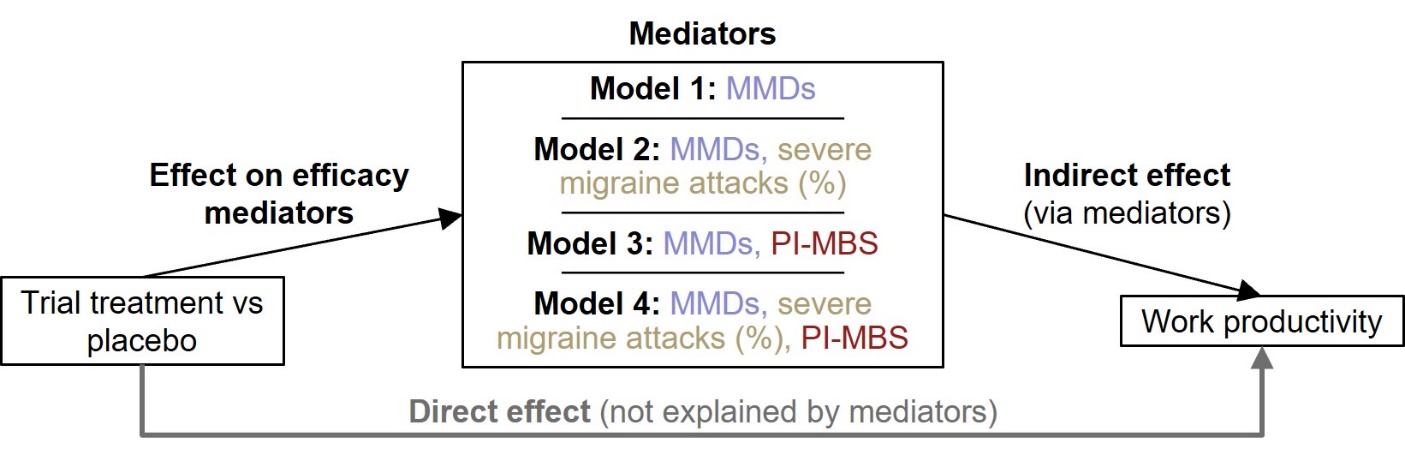
**

MMDs, monthly migraine days; PI-MBS, patient-identified most bothersome symptom.

**Figure S2.** Percent of interviewed patients endorsing themes cohering on the impact of migraine on work-related activities

**Table S2.** All absenteeism and presenteeism models

| **Model** | **AIC** | **BIC** |
| --- | --- | --- |
| **Absenteeism** |  |  |
| Quadratic model | 34950.0 | 34981.3 |
| Linear model | 34956.3 | 34981.3 |
| **Presenteeism** |  |  |
| Quadratic model | 40088.1 | 40119.8 |
| Linear model | 40191.4 | 40216.7 |

AIC, Akaike’s Information Criteria; BIC, Bayesian Information Criteria. Lower values indicate better fit.

**Table S3.** Best-fit model for absenteeism and presenteeism

| **Variable** | **Coefficient (SE)** | **df** | ***t*-value** | ***p*-value** |
| --- | --- | --- | --- | --- |
| **Absenteeism^†^** |  |  |  |  |
| Intercept | 2.15 (0.96) | 1197.8 | 2.25 | 0.025 |
| MMDs | 1.04 (0.07) | 2832.9 | 14.35 | <0.001 |
| **Presenteeism^‡^** |  |  |  |  |
| Intercept | 11.07 (1.70) | 2145.7 | 6.51 | <0.001 |
| MMDs | 6.18 (0.29) | 3743.9 | 21.29 | <0.001 |
| MMDs^2^ | -0.12 (0.01) | 3637.7 | -10.19 | <0.001 |

^†^3847 observations. ^‡^3793 observations. *N* = 656 patients. df, degrees of freedom; MMDs, monthly migraine days; SE, standard error.

**Figure S3.** Eptinezumab treatment effect in addition to MMDs for monthly (A) absenteeism and (B) presenteeism


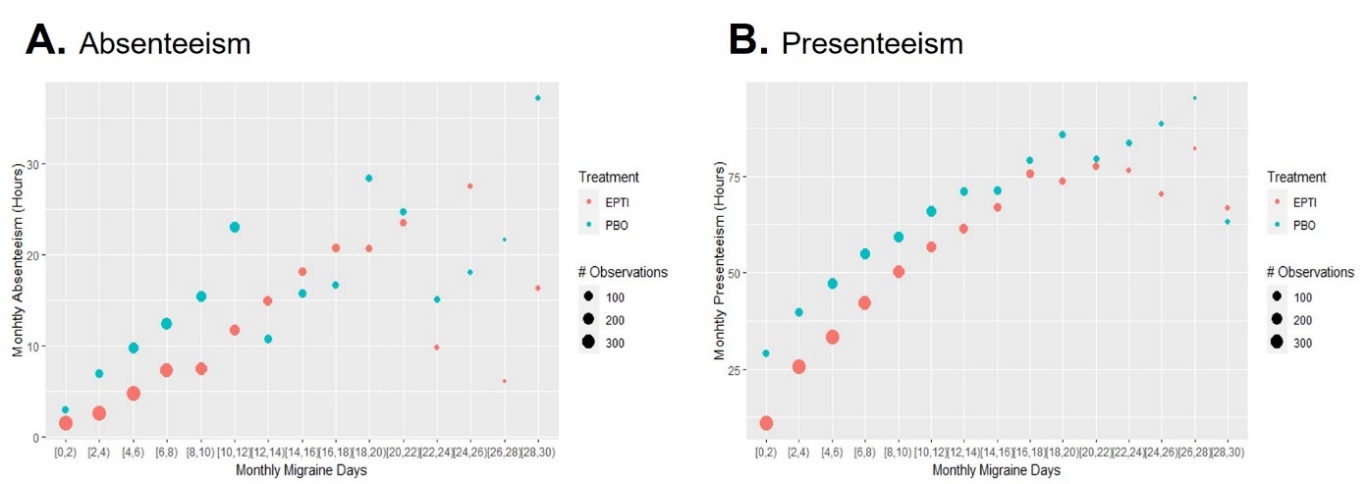


EPTI, eptinezumab; MMDs, monthly migraine days; PBO, placebo. Patients self-reported their monthly hours of absenteeism and presenteeism.

**Table S4.** Best-fit models for the eptinezumab treatment effect in addition to MMD reduction

| **Variable** | **Coefficient (SE)** | **df** | ***t*-value** | ***p*-value** |
| --- | --- | --- | --- | --- |
| **Absenteeism^†^** |  |  |  |  |
| Intercept | 6.04 (1.43) | 897.3 | 4.21 | <0.001 |
| MMDs | 0.89 (0.08) | 1914 | 10.63 | <0.001 |
| Eptinezumab | -4.81 (1.41) | 625.1 | -3.41 | <0.001 |
| **Presenteeism^‡^** |  |  |  |  |
| Intercept | 25.04 (2.51) | 1290 | 9.97 | <0.001 |
| MMDs | 4.78 (0.33) | 3026.8 | 14.56 | <0.001 |
| MMDs^2^ | -0.09 (0.01) | 2979 | -6.96 | <0.001 |
| Eptinezumab | -10.78 (2.27) | 656.6 | -4.76 | <0.001 |

^†^3224 observations. ^‡^3184 observations. *N* = 656 patients. df, degrees of freedom; MMDs, monthly migraine days; SE, standard error.

**Table S5.** Analysis of self-reported monthly absenteeism and presenteeism hours in the HFEM+CM population

|  | **Baseline** | | **Change from Baseline** | | **Comparison to Placebo** | | |
| --- | --- | --- | --- | --- | --- | --- | --- |
| **Treatment** | ***N*** | **Mean (SD)** | ***N*^†^** | **Mean (SE)** | **Difference** | **95% CI** | ***p*-value** |
| **Absenteeism^‡^** |  |  |  |  |  |  |  |
| Placebo | 192 | 19.7 (29.7) | 185 | -3.3 (2.20) |  |  |  |
| Eptinezumab 100 mg | 167 | 18.3 (30.3) | 163 | -10.0 (2.21) | -6.7 | (-10.2, -3.2) | <0.001 |
| Eptinezumab 300 mg | 184 | 18.9 (29.7) | 179 | -9.6 (2.17) | -6.3 | (-9.7, -2.9) | <0.001 |
| Eptinezumab pooled | 351 | 18.6 (30.0) | 342 | -9.9 (1.99) | -6.5 | (-9.4, -3.5) | <0.001 |
| Eptinezumab responders^§^ | 154 | 18.8 (28.3) | 151 | -15.3 (2.23) | -11.4 | (-14.9, -7.9) | <0.001 |
| **Presenteeism^¶^** |  |  |  |  |  |  |  |
| Placebo | 187 | 80.3 (34.6) | 182 | -12.7 (3.65) |  |  |  |
| Eptinezumab 100 mg | 162 | 77.5 (37.3) | 158 | -33.7 (3.69) | -20.9 | (-27.2, -14.7) | <0.001 |
| Eptinezumab 300 mg | 181 | 80.3 (35.4) | 178 | -34.2 (3.58) | -21.4 | (-27.5, -15.4) | <0.001 |
| Eptinezumab pooled | 343 | 79.0 (36.3) | 336 | -33.7 (3.26) | -21.2 | (-26.4, -15.9) | <0.001 |
| Eptinezumab responders^§^ | 152 | 79.6 (39.8) | 150 | -51.4 (3.46) | -37.6 | (-43.4, -31.8) | <0.001 |

**^†^**Number of patients with ≥1 measurement. **^‡^**2675 observations. ^§^Responders are defined as patients reaching ≥50% reduction from baseline in MMDs for each month in the 3-month interval (weeks 1‒12). **^¶^**2602 observations.

CI, confidence interval; CM, chronic migraine; HFEM, high-frequency episodic migraine; MMDs, monthly migraine days; SD, standard deviation; SE, standard error. Analysis was of the full analysis set and utilized a mixed model for repeated measures.

**Table S6.** Analysis of self-reported monthly absenteeism and presenteeism hours in the United States

|  | **Baseline** | | **Change from Baseline** | | **Comparison to Placebo** | | |
| --- | --- | --- | --- | --- | --- | --- | --- |
| **Treatment** | ***N*** | **Mean (SD)** | ***N*^†^** | **Mean (SE)** | **Difference** | **95% CI** | ***p*-value** |
| **Absenteeism^‡^** |  |  |  |  |  |  |  |
| Placebo | 218 | 19.9 (31.1) | 210 | -1.0 (1.80) |  |  |  |
| Eptinezumab 100 mg | 196 | 17.7 (30.0) | 192 | -8.7 (1.82) | -7.7 | (-11.0, -4.4) | <0.001 |
| Eptinezumab 300 mg | 209 | 18.5 (29.9) | 204 | -8.3 (1.76) | -7.3 | (-10.5, -4.0) | <0.001 |
| Eptinezumab pooled | 405 | 18.1 (29.9) | 396 | -8.6 (1.57) | -7.5 | (-10.3, -4.7) | <0.001 |
| Eptinezumab responders^§^ | 185 | 18.2 (28.3) | 182 | -13.4 (1.82) | -12.1 | (-15.4, -8.8) | <0.001 |
| **Presenteeism^¶^** |  |  |  |  |  |  |  |
| Placebo | 212 | 80.0 (37.5) | 206 | -12.6 (3.28) |  |  |  |
| Eptinezumab 100 mg | 191 | 78.6 (39.6) | 187 | -35.0 (3.33) | -22.4 | (-28.4, -16.4) | <0.001 |
| Eptinezumab 300 mg | 206 | 82.6 (37.2) | 203 | -34.7 (3.19) | -22.0 | (-27.9, -16.2) | <0.001 |
| Eptinezumab pooled | 397 | 80.7 (38.4) | 390 | -34.6 (2.87) | -22.2 | (-27.3, -17.1) | <0.001 |
| Eptinezumab responders^§^ | 183 | 80.5 (41.4) | 181 | -52.1 (3.10) | -38.6 | (-44.2, -33.0) | <0.001 |

**^†^**Number of patients with ≥1 measurement. **^‡^**3088 observations. ^§^Responders are defined as patients reaching ≥50% reduction from baseline in MMDs for each month in the 3-month interval (weeks 1‒12). **^¶^**3005 observations.

CI, confidence interval; MMDs, monthly migraine days; SD, standard deviation; SE, standard error. Analysis was of the full analysis set and utilized a mixed model for repeated measures.

**Table S7.** Analysis of self-reported monthly absenteeism and presenteeism hours in the United Kingdom

|  | **Baseline** | | **Change from Baseline** | | **Comparison to Placebo** | | |
| --- | --- | --- | --- | --- | --- | --- | --- |
| **Treatment** | ***N*** | **Mean (SD)** | ***N*^†^** | **Mean (SE)** | **Difference** | **95% CI** | ***p*-value** |
| **Absenteeism^‡^** |  |  |  |  |  |  |  |
| Placebo | 218 | 18.1 (28.3) | 210 | -0.9 (1.64) |  |  |  |
| Eptinezumab 100 mg | 196 | 16.1 (27.4) | 192 | -8.0 (1.66) | -7.1 | (-10.1, -4.0) | <0.001 |
| Eptinezumab 300 mg | 209 | 16.9 (27.3) | 204 | -7.5 (1.60) | -6.6 | (-9.6, -3.7) | <0.001 |
| Eptinezumab pooled | 405 | 16.5 (27.3) | 396 | -7.8 (1.43) | -6.8 | (-9.4, -4.3) | <0.001 |
| Eptinezumab responders^§^ | 185 | 16.6 (25.8) | 182 | -12.3 (1.66) | -11.0 | (-14.0, -8.0) | <0.001 |
| **Presenteeism^¶^** |  |  |  |  |  |  |  |
| Placebo | 212 | 73.0 (34.2) | 206 | -11.5 (2.99) |  |  |  |
| Eptinezumab 100 mg | 191 | 71.7 (36.2) | 187 | -32.0 (3.03) | -20.4 | (-25.9, -15.0) | <0.001 |
| Eptinezumab 300 mg | 206 | 75.3 (33.9) | 203 | -31.6 (2.91) | -20.1 | (-25.5, -14.7) | <0.001 |
| Eptinezumab pooled | 397 | 73.6 (35.0) | 390 | -31.6 (2.62) | -20.2 | (-24.9, -15.6) | <0.001 |
| Eptinezumab responders^§^ | 183 | 73.5 (37.7) | 181 | -47.5 (2.83) | -35.2 | (-40.3, -30.1) | <0.001 |

**^†^**Number of patients with ≥1 measurement. **^‡^**3088 observations. ^§^Responders are defined as patients reaching ≥50% reduction from baseline in MMDs for each month in the 3-month interval (weeks 1‒12). **^¶^**3005 observations.

CI, confidence interval; MMDs, monthly migraine days; SD, standard deviation; SE, standard error. Analysis was of the full analysis set and utilized a mixed model for repeated measures.

**Table S8.** Analysis of self-reported monthly absenteeism and presenteeism hours in Germany

|  | **Baseline** | | **Change from Baseline** | | **Comparison to Placebo** | | |
| --- | --- | --- | --- | --- | --- | --- | --- |
| **Treatment** | ***N*** | **Mean (SD)** | ***N*^†^** | **Mean (SE)** | **Difference** | **95% CI** | ***p*-value** |
| **Absenteeism^‡^** |  |  |  |  |  |  |  |
| Placebo | 218 | 15.6 (24.4) | 210 | -0.8 (1.41) |  |  |  |
| Eptinezumab 100 mg | 196 | 13.9 (23.6) | 192 | -6.9 (1.43) | -6.1 | (-8.7, -3.5) | <0.001 |
| Eptinezumab 300 mg | 209 | 14.5 (23.5) | 204 | -6.5 (1.38) | -5.7 | (-8.3, -3.2) | <0.001 |
| Eptinezumab pooled | 405 | 14.2 (23.5) | 396 | -6.7 (1.24) | -5.9 | (- 8.1, -3.7) | <0.001 |
| Eptinezumab responders^§^ | 185 | 14.3 (22.2) | 182 | -10.6 (1.43) | -9.5 | (-12.1, -6.9) | <0.001 |
| **Presenteeism^¶^** |  |  |  |  |  |  |  |
| Placebo | 212 | 62.9 (29.5) | 206 | -9.9 (2.50) |  |  |  |
| Eptinezumab 100 mg | 191 | 61.8 (31.1) | 187 | -27.5 (2.61) | -17.6 | (-22.3, -12.9) | <0.001 |
| Eptinezumab 300 mg | 206 | 64.9 (29.2) | 203 | -27.2 (2.5l) | -17.3 | (-21.9, -12.7) | <0.001 |
| Eptinezumab pooled | 397 | 63.4 (30.1) | 390 | -27.2 (2.25) | -17.4 | (-21.5, -13.4) | <0.001 |
| Eptinezumab responders^§^ | 183 | 63.3 (32.5) | 181 | -40.9 (2.43) | -30.3 | (-34.7, -25.9) | <0.001 |

**^†^**Number of patients with ≥1 measurement. **^‡^**3088 observations. ^§^Responders are defined as patients reaching ≥50% reduction from baseline in MMDs for each month in the 3-month interval (weeks 1‒12). **^¶^**3005 observations.

CI, confidence interval; MMDs, monthly migraine days; SD, standard deviation; SE, standard error. Analysis was of the full analysis set and utilized a mixed model for repeated measures.

**Table S9.** Sensitivity analysis of self-reported monthly absenteeism and presenteeism hours using the WPAI:M captured in DELIVER

|  | **Baseline** | | **Change from Baseline** | | **Comparison to Placebo** | | |
| --- | --- | --- | --- | --- | --- | --- | --- |
| **Treatment** | ***N*** | **Mean (SD)** | ***N*^†^** | **Mean (SE)** | **Difference** | **95% CI** | ***p*-value** |
| **Absenteeism^‡^** |  |  |  |  |  |  |  |
| Placebo | 227 | 21.1 (53.5) | 222 | -1.7 (1.93) | NA | NA |  |
| Eptinezumab 100 mg | 206 | 14.5 (23.5) | 203 | -6.8 (1.95) | -5.2 | (-8.6, -1.7) | 0.003 |
| Eptinezumab 300 mg | 223 | 16.0 (34.1) | 218 | -6.8 (1.89) | -5.1 | (-8.5, -1.8) | 0.003 |
| Eptinezumab pooled | 429 | 15.3 (29.5) | 421 | -6.9 (1.71) | -5.2 | (-8.1, -2.2) | <0.001 |
| Eptinezumab responders^§^ | 197 | 13.6 (20.6) | 196 | -11.9 (1.96) | -9.9 | (-13.3, -6.4) | <0.001 |
| **Presenteeism^¶^** |  |  |  |  |  |  |  |
| Placebo | 212 | 72.4 (48.1) | 206 | -10.1 (3.47) | NA | NA |  |
| Eptinezumab 100 mg | 191 | 68.8 (45.2) | 187 | -28.3 (3.53) | -18.1 | (-24.4, -11.8) | <0.001 |
| Eptinezumab 300 mg | 206 | 70.7 (52.8) | 203 | -26.7 (3.40) | -16.6 | (-22.7, -10.4) | <0.001 |
| Eptinezumab pooled | 397 | 69.7 (49.2) | 390 | -27.3 (3.07) | -17.3 | (-22.6, -11.9) | <0.001 |
| Eptinezumab responders^§^ | 183 | 66.1 (51.2) | 181 | -41.9 (3.41) | -31.0 | (-37.0, -24.9) | <0.001 |

**^†^**Number of patients with ≥1 measurement. **^‡^**3569 observations. Absenteeism was calculated by multiplying the results of WPAI:M Question 2 (“During the past seven days, how many hours did you miss from work because of your health problems?”) by 4 to obtain monthly hours lost to absenteeism. ^§^Responders are defined as patients reaching ≥50% reduction from baseline in MMDs for each month in the 3-month interval (weeks 1‒12). **^¶^**3034 observations. Presenteeism was calculated with the following formula: Q4×(Q5/10)×4 with Q4 being the response to “During the past seven days, how many hours did you actually work?” and Q5 being a 10-point-scale response to the following question: “During the past seven days, how much did your health problems affect your productivity while you were working?”

CI, confidence interval; MMDs, monthly migraine days; SD, standard deviation; SE, standard error; WPAI:M, migraine-specific Work Productivity and Activity Impairment. Analysis was of the full analysis set and utilized a mixed model for repeated measures.

**Figure S4.** Productivity gains following eptinezumab infusion in (A) the United States, (B) the United Kingdom, and (C) Germany

N = 656 patients. Value of productivity gains = change from baseline in monthly hours in pooled eptinezumab group × 3 months × [average hourly wage rate]. In 2021, the average hourly wage rate was (A) 28.01 USD [1]; (B) 14.48 GBP [2]; (C) 23.56 EUR [3]. EUR, euro; GBP, pound sterling; USD, United States dollar. The costing hours affected by presenteeism were assumed to be at a 100% productivity level. The time period was 12 weeks (3 months) during which patients received one eptinezumab infusion.

[1] U.S. Bureau of Labor Statistics. May 2021 National Occupational Employment and Wage Estimates. Available at: https://www.bls.gov/oes/current/oes_nat.htm. Last modified: Mar. 31, 2022. Accessed Mar. 23, 2023.

[2] Office for National Statistics. EARN01: Average weekly earnings. Available at: https://www.ons.gov.uk/employmentandlabourmarket/peopleinwork/earningsandworkinghours/datasets/averageweeklyearningsearn01. Release date: Mar. 14, 2023. Accessed Mar. 23, 2023.

[3] Statistisches Bundesamt (Destatis; German Federal Statistical Office). Available at: https://www.destatis.de/EN/Themes/Labour/Earnings/Earnings-Earnings-Differences/current-economic-activity.html. 2023. Accessed Mar. 23, 2023.

**Figure S5.** Path analysis exploring the effects of treatment on reducing monthly hours of absenteeism: (A) with change in MMDs as the mediator; (B) with changes in MMDs and percent of migraine attacks with severe pain as mediators; (C) with changes in MMD and PI-MBS as mediators; (D) with changes in all three variables as mediators

Δ, change from baseline in [variable]; MMDs, monthly migraine days; *N*, number of patients; Obs, number of observations; PI-MBS, patient-identified most bothersome symptom. ****p* < 0.001; ***p* < 0.01; **p* < 0.05.

**Figure S6.** Path analysis exploring the effects of treatment on reducing monthly hours of presenteeism: (A) with change in MMDs as the mediator; (B) with changes in MMDs and percent of migraine attacks with severe pain as mediators; (C) with changes in MMD and PI-MBS as mediators; (D) with changes in all three variables as mediators

**
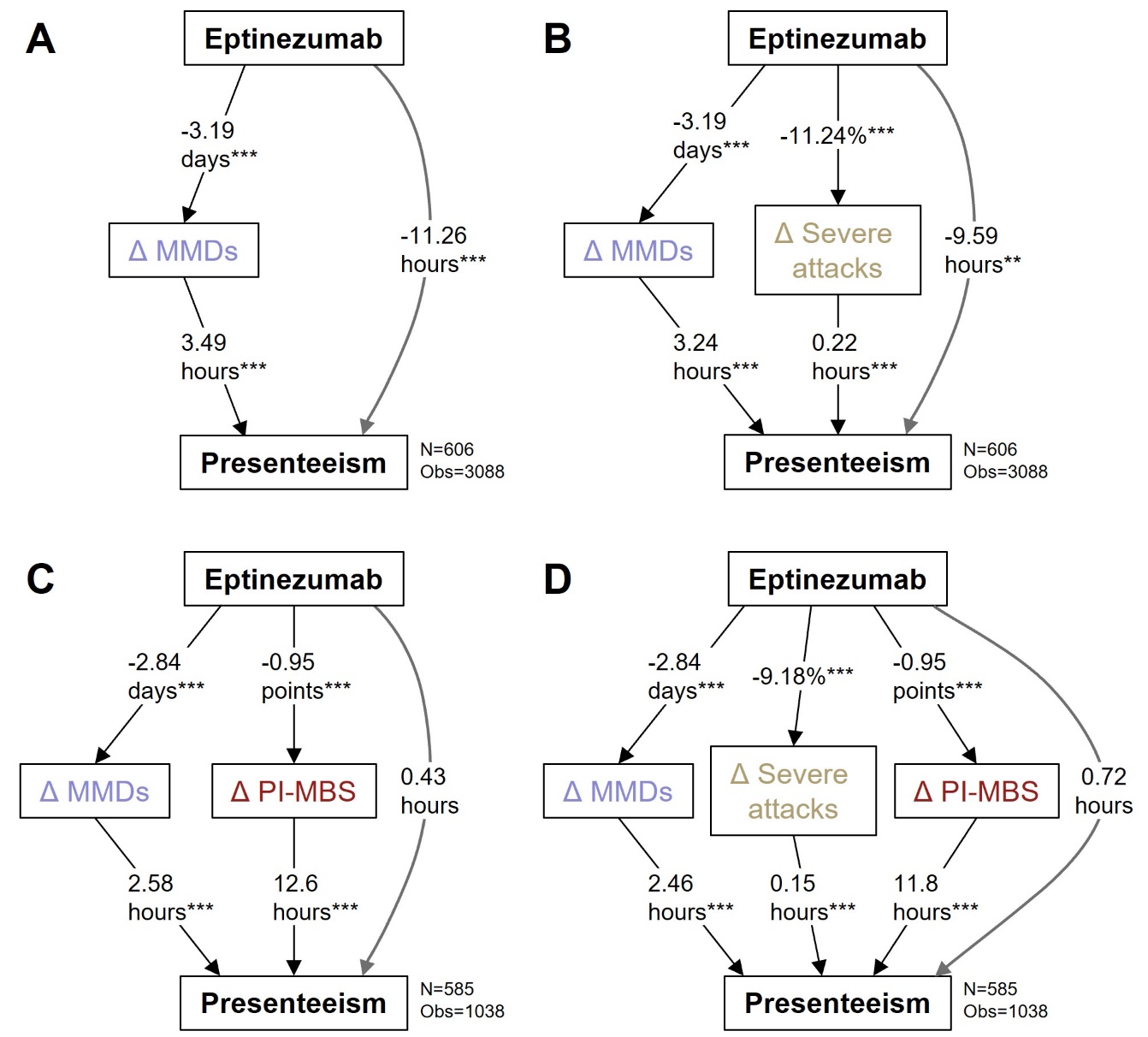
**

Δ, change from baseline in [variable]; MMDs, monthly migraine days; *N*, number of patients; Obs, number of observations; PI-MBS, patient-identified most bothersome symptom. ****p* < 0.001; ***p* < 0.01; **p* < 0.05.

**Figure S7.** Direct versus indirect treatment effect for A) absenteeism and B) presenteeism, including all direct effects


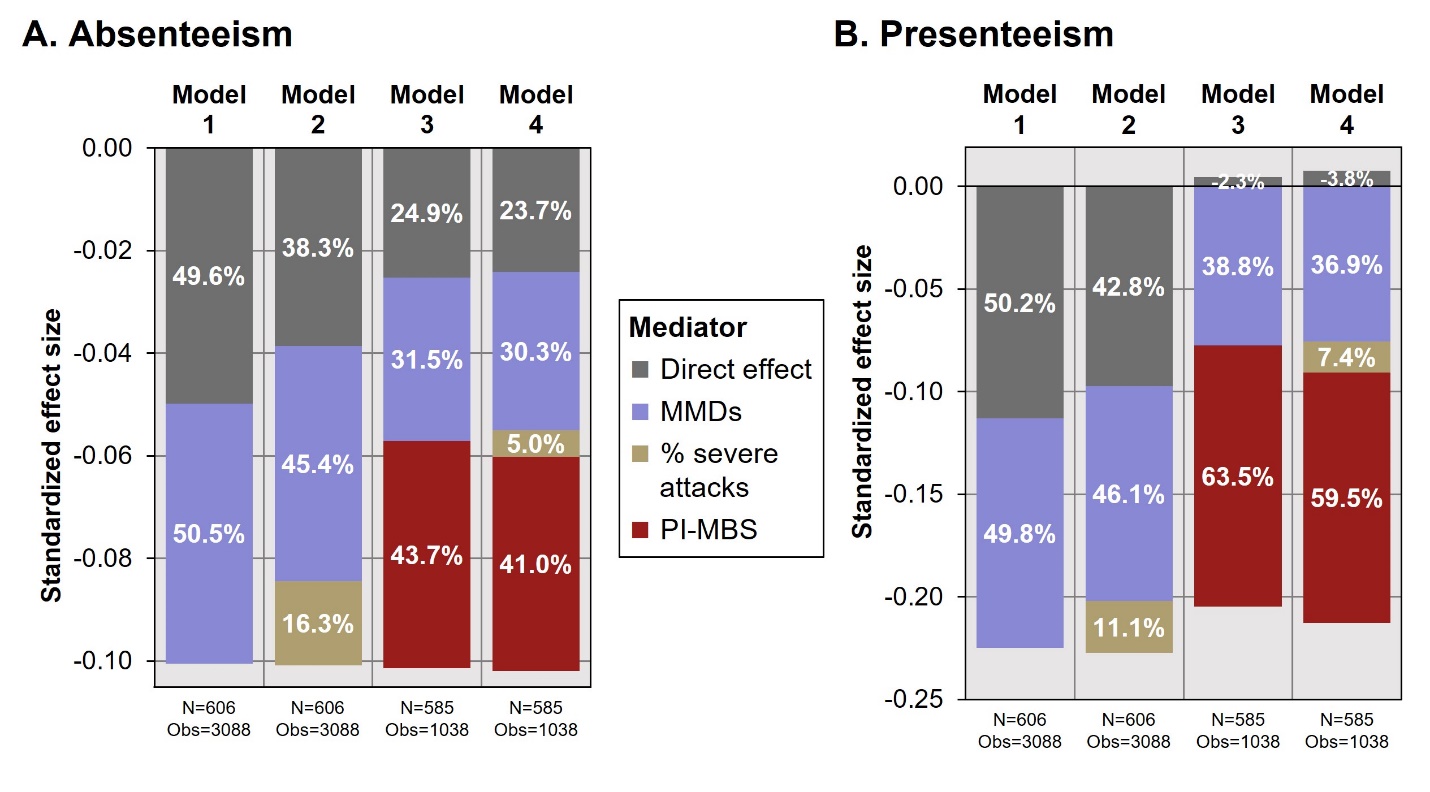


Due to rounding, some percentages may not add up to 100%.

(B) Presenteeism Models 3 and 4: The direct effect was small (<5%), negative, and not significant (p-value < 0.05) meaning that the direct effect could not be proven to be different from 0 and that the sign of the coefficient should not be overly interpreted.

For presenteeism Models 3 and 4, the indirect effects may fully account for the relationship between the treatment and the outcome, resulting in an estimated direct effect that could be influenced by random fluctuations and lead to an artificially small or even negative value.

MMDs, monthly migraine days; PI-MBS, patient-identified most bothersome symptom.

**Table S10.** Impact of country on eptinezumab’s treatment effect.

| **Model** | **AIC** | **BIC** |
| --- | --- | --- |
| **Absenteeism** |  |  |
| With country effect | 31401.5 | 31744.3 |
| Without country effect | 31448.3 | 31723.8 |
| **Presenteeism** |  |  |
| With country effect | 29276.0 | 29609.5 |
| Without country effect | 29323.7 | 29591.8 |

AIC, Akaike Information Criterion; BIC, Bayesian Information Criterion.
